# SmartTags: IoT Product Passport for Circular Economy Based on Printed Sensors and Unique Item-Level Identifiers

**DOI:** 10.3390/s19030586

**Published:** 2019-01-30

**Authors:** Nenad Gligoric, Srdjan Krco, Liisa Hakola, Kaisa Vehmas, Suparna De, Klaus Moessner, Kristoffer Jansson, Ingmar Polenz, Rob van Kranenburg

**Affiliations:** 1 DunavNET, 21000 Novi Sad, Serbia; srdjan.krco@dunavnet.eu; 2 VTT Technical Research Centre, 02150 Espoo, Finland; liisa.hakola@vtt.fi (L.H.); kaisa.vehmas@vtt.fi (K.V.); 3 Institute for Communication Systems, University of Surrey, Guildford GU2 7XH, UK; s.de@surrey.ac.uk (S.D.); k.moessner@surrey.ac.uk (K.M.); 4 UpCode Ltd, 66560 Vaasa, Finland; kristoffer.jansson@upcode.fi; 5 Durst Phototechnik Digital Technology GmbH, 9900 Lienz, Austria; ingmar.polenz@durst-group.com; 6 Resonance Design, 5046KK Tilburg, The Netherlands; kranenbu@xs4all.nl

**Keywords:** IoT, circular economy, product passport, printed sensors, functional ink, ontology, barcode, GS1

## Abstract

In this paper, we present a method that facilitates Internet of Things (IoT) for building a product passport and data exchange enabling the next stage of the circular economy. SmartTags based on printed sensors (i.e., using functional ink) and a modified GS1 barcode standard enable unique identification of objects on a per item-level (including Fast-Moving Consumer Goods—FMCG), collecting, sensing, and reading of parameters from environment as well as tracking a products’ lifecycle. The developed ontology is the first effort to define a semantic model for dynamic sensors, including datamatrix and QR codes. The evaluation of decoding and readability of identifiers (QR codes) showed good performance for detection of sensor state printed over and outside the QR code data matrix, i.e., the recognition ability with image vision algorithm was possible. The evaluation of the decoding performance of the QR code data matrix printed with sensors was also efficient, i.e., the QR code ability to be decoded with the reader after reversible and irreversible process of ink (dis)appearing was preserved, with slight drop in performance if ink density is low.

## 1. Introduction

The Internet of Things (IoT) greatest potential is the promise of scaling huge numbers to billions of items and devices. The basic level of identification of an item is to be able to look up its specific properties, usually in terms of description and branding information. There are already some widely-adopted identification standards such as those under the GS1 organization [1] that provides a mechanism to create globally recognized identifiers for products. However, currently these identifiers are mostly used to recognize products at the Stock Keeping Unit (SKU) level, which leaves two key aspects to transform a product into a digital asset unsolved: on the one hand, how to identify the specific item itself, not only the product, and on the other hand, objects connected to Internet will have a unique lifecycle and can be shared among different stakeholders and different systems. 

Further to this, item-level identification can serve as a disruptive innovation, launching new business models for the circular economy (CE), that will be worth $4.5 trillion by 2030 [2]. In this vision, the Internet of Things is the key enabler: IoT and circular economy are both about a complete system reinvention, and they are both about smart, informed management of assets. In the report on the circular economy potential [3], Intelligent Assets establishes an interplay between “value drivers” of a circular model, and the potential benefits offered by a network of connected devices and information which ensures that businesses around the world can make the right decisions to eradicate waste and use resources effectively. Pairing circular economy principles with the information generated by smart devices creates a fertile ground for innovation that could enable acceleration of the linear economy of today. Products will communicate with users, collectors and remanufacturers to ensure they are returned and reused after their first life cycle. Additionally, condition monitoring of sensitive goods during transport, storage, and use will expand product lifetime [4]. The trends outlined above demand transparency in supply chain and federated identities that expose wishes to service providers and product developers without being immediately identifiable. A product passport is a key enabler in this new situation on real time updates and insight by all stakeholders from the producer, the transport organizers, the retailer, and consumer. 

We see intelligent devices as one of the enablers of such scenarios towards a society in which value creation is increasingly decoupled from consumption of finite resources. To pursue this goal, having unique identity on item-level is crucial to be able to identify attributes of the product passport that are reusable and to monitor its changes during the lifecycle. In addition, it is required to develop a mechanism that will enable context detection which influences these changes (e.g., in which conditions the resource is being used, for how long, what is its current state, etc.). 

In this paper we have proposed SmartTag technology built from combination of printed sensors and QR codes with unique item-level IDs able to sense predefined changes in the environment. Printed sensors are made from functional ink that reacts on temperature and luminosity (it is possible to sense other parameters, but we have focused on these two in this research).

We have tackled several research problems that can be addressed using proposed SmartTags technology. The first problem is the unique identification of products on an item-level, based on modification of the ISO/IEC 18004:2000 standard to support the extended information embedded in the QR code. The second contribution of the paper is methodology for encoding printed sensors in identifiers, as well as methodology for development of the printed sensors based on functional ink that reacts upon different environmental conditions (in this case temperature and luminosity). More specifically, thermochromic and photochromic colorant systems were developed, both reversibly and irreversibly, colorize-able by temperature and visible light (VIS). Further to this, the SmartTag technology’s unique identification is supported with the ability to sense conditions from the product’s environment on per item-level leveraging functional disappearing and appearing ink. The preservation of qualitative information embedded inside a QR code after its modification requires identification of the functional ink type requirements which are also provided. To the best of our knowledge this is the first attempt to identify the problem and specify requirements for such a system. The method for formulation of the sensors made with two different ink types is proposed and evaluated for the SmartTags. Thermochromic inks are not widely available for inkjet printing, that is an essential printing method when printing individual tags. 

The data exchange leverages on the sensing capability of the identifiers tagged on entities, which when used together with other (meta) information creates wider knowledge about the context. The circular economy is driven by the data exchange which requires a unified semantic ontology. In the moment of writing this paper, the only available ontology for circular economy was presented in [5], where the author proposed linked spatial data for a textile use case. We have extended this study with the SmartTag semantic ontology and ontology for the circular economy. Based on a review of the existing literature presented in Section 2 below, we believe that the developed SmartTag ontology is the first effort to define a semantic model that brings together a dynamical sensor based datamatrix and QR codes.

The remainder of this paper is organized in the following manner. In Section 2, state of the art on related work: unique item identification, printed sensors, and ontologies is presented. In Section 3, the overall methodology for developing, encoding, and evaluating printed sensors based on functional ink is described. The performance evaluation of printed photochromic and thermochromic sensors is presented in Section 4. The circular economy ontology and semantic model for the SmartTag as well as example of the ontology for evaluated use cases are provided in Section 5. In Section 6, a discussion is provided. The paper is concluded in Section 7. The standardization potential is explained is Section 8.

## 2. State of the Art

This section provides an overview of relevant state of the art on contextual codes, semantic data models and data standards for smart object and device modelling, drafted from EU projects in the IoT area and from the wider global research community. Semantic models describing the associated topics of observation and measurement data are also reviewed here. This is used to define and build the framework as a starting point of SmartTag modelling.

### 2.1. Identification with Contextual QR Code

Efforts to automatically identify and attach information to individually tagged products, including prototype applications, date from 1999 when employees at Xerox Parc were augmenting books and documents with radio-frequency identification (RFID) tags and linking them to associated services [6]. More recently, the prevalence of 1-D and 2-D codes for conveying enhanced information has been applied to proof-of-concept applications such as the mobile phone-based Allergy Assistant [7] that recognizes a product information through its barcode and associates it with a person’s predefined profile (containing his/her known allergies) to provide on-screen cues as to whether the product is suitable for consumption. Other approaches feature specific user interaction that shows different information and services dependent upon the rotation of the phone while scanning the barcode [8]. Currently, already existing tags focus mainly on item identification at the product level based on following existing different encoding mechanisms:

- Barcodes: the most widespread technology, attached to almost every product in the market (1D Barcodes) and progressively including more sophisticated ones (2D codes, such as QR-Codes and data matrix codes). The later encodes information based on certain geometrical figures and spaces between them. An optical reader is needed to decode the values.
- Electronic Tags: by using small integrated circuits, these tags can store information in a digital format that can be read by a compatible scanner. The most used ones, with varying underlying communication technologies, are near-field communication (NFC)/RFID.

While the above approaches have used static encoded information in the codes, Reference [9] has looked at including the context of the scan to present dynamic information. The developed application merges the decoded QR code information along with context information obtained from the scanning mobile phone, such as the device, user’s profile, location, time, etc. However, the information coded inside the QR code remains static, unlike the dynamic SmartTags we are proposing.

### 2.2. Related Work on Printed Sensors

Printed sensors are (sensing) devices manufactured using printing methods. Typical benefits of using printing technologies include the possibility to use flexible substrates resulting in thin and light-weight devices, more affordable, and using simpler infrastructure than, for example, the semiconductor industry. This raises the possibility to design innovative and novel products for markets not yet addressed by the electronics industry. Since (printed) sensors have much simpler structures than displays or logic circuits, the manufacturing learning curve is less steep compared to many other printed electronic applications. The most intensively evolving types of printed sensors include biosensors, capacitive sensors, piezoresistive sensors, piezoelectric sensors, photodetectors, temperature sensors, humidity sensors, and gas sensors [10].

Indicators are sensors based on optical readings, such as color change. In the simplest form they are functional inks reacting to environmental conditions, such as temperature or lighting. The most popular and readily available functional ink technologies are thermochromic (changing color with temperature) and photochromic (changing color with ultraviolet/sun light). Other commercially available ink technologies include invisible fluorescent inks (seen under UV or IR light), phosphorescent inks (glow in the dark after exposure to a source of light), hydrochromic inks (changing color after contact with water), and touch and smell inks (aroma released when rubbed with a finger), among others. More complex indicators are actual systems that involve multiple processing steps, such as activation and encapsulation. Most often, the information is displayed by immediate visual changes, e.g., different color intensities or the diffusion of a dye along the indicator geometry. A distinct feature of indicators is the type of information involved, which is qualitative or semi-quantitative in nature. Indicators with applications to food packaging are time temperature indicators, oxygen and integrity indicators, and freshness indicators. Sensors can be printed on different materials including paper, and foil [11], and are usually used in products for mass consumption such as food packages [12,13,14]. 

### 2.3. Related Work on Ontologies

**Figure 1:**
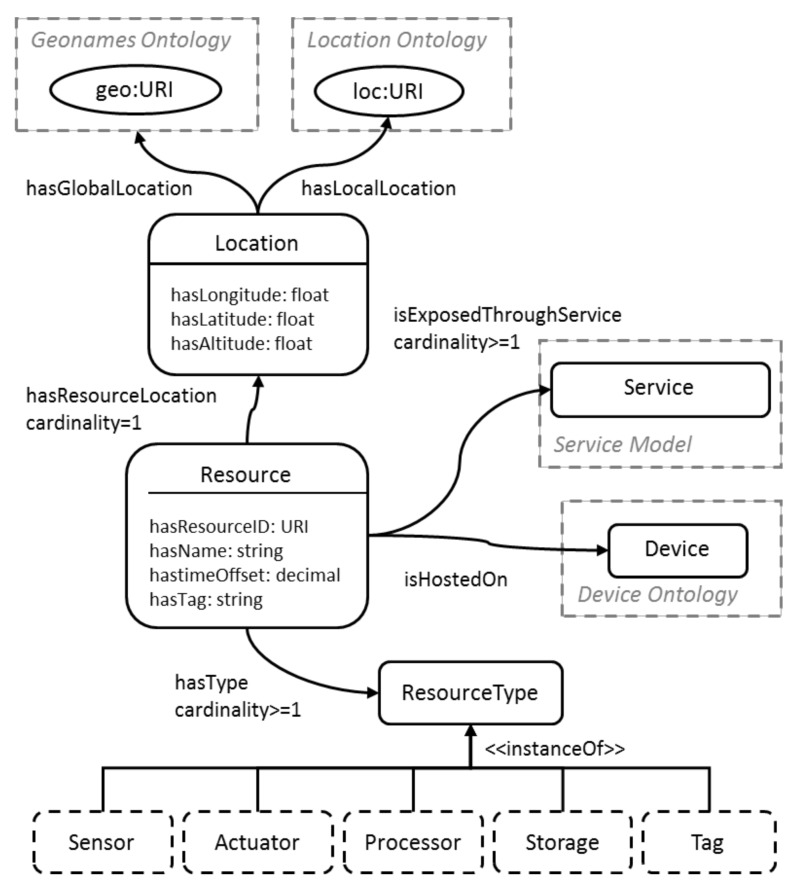
Internet of Things Architecture (IoT-A) Resource Model.

Semantic for the IoT specific elements, as well as functionalities and associated models with metadata, has been previously presented in several initiatives including EU projects and research. A detailed survey of the semantic models in the IoT domain is available in [15]. In the Internet of Things-Architecture (IoT-A) project, an IoT domain model [16] is defined, specifying the trio of entity, resource, and service as the main elements of the IoT. Inter-relationships between these three concepts, as well as further specializations, were also defined. IoT-A also defined the corresponding semantic model [17] for entities, resources, and services, with resources forming the software representation of device functionalities. Relevant to the work proposed in this paper is the resource model, which defines resource types (e.g., sensor/actuator/gateway node), corresponding device location and further specification of the resource capabilities through the linked service model. Resource location is specified through geographical coordinates, a GeoNames [18] ontology URI and also as a datatype property link to a local location ontology instance, which models detailed relevant orientations of rooms and buildings within a campus [19]. A simplified version of the IoT-A resource model [17] is shown in Figure 1.

**Figure 2:**
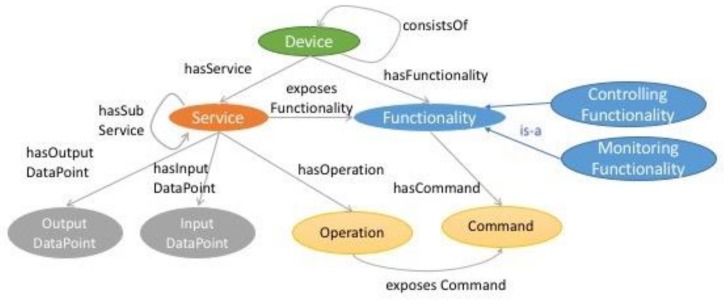
Main concept of oneM2M Base ontology, adapted from [22].

A device model is also proposed in [20] and in [21], where resources are modelled as the computational element of a device. These are further classified into on-device or network resource, to capture various possible events in an IoT environment. The oneM2M Base ontology [22] from the oneM2M standards initiative, is shown in Figure 2. It models, as its top-most element, a Thing, which can represent an entity in the oneM2M system. Its sub-element is the Device class, which specifies something that can interact electronically with the environment. A device is modelled in terms of its functionalities (i.e., capabilities), with cardinality ≥1, which are exposed to the network through services. These modelled capabilities are associated with either sensing aspects (measuringFunctionality) or actuation aspects (controllingFunctionality).

An extended semantic model has been built in the COSMOS project [23] which spans from the topmost abstractions, the Virtual Entities (VEs) and the Group Virtual Entities (GVEs), down to the Physical Entities (PEs), and the underlying IoT services including their endpoint parameters. This part of the ontology also covers the VE and GVE location definition, providing the means to link it either to a GeoNames based location, a user defined location ontology or external location service for mobile VEs. Another part of the semantic model is dedicated to the social relations among the VEs, a concept which was explored as part of the COSMOS project. It models the links among VEs and introduces concept such as trust and reputation to support information sharing and resource reuse. The third part of the semantic model consists of domain ontologies. While the core part and the social relation part of the semantic model are generic and domain independent, domain ontologies are used to link the core entities to application and domain specific descriptions. For example, if the data type (e.g., integer, double, string etc.) of a service endpoint is covered by the core ontology, its meaning, which is in COSMOS also a part of the description, is described using a domain ontology (e.g., temperature, traffic level, blood pressure etc.).

A number of semantic appliance models have been defined in the smart home domain. Prominent among them is the DogOnt ontology [24], which describes homes in a device and network-agnostic way. It specifies both appliances that can be controlled as well as the structural elements of the home, such as a room or garden. In addition, also defined in the ontology are properties that associate the modelled appliances to their offered functionality. The modelled functionalities include those that relate to notification, query and control, current state and communication components. The Smart Appliances REFerence (SAREF) ontology [25] abstracts away the inherent heterogeneity of communication protocols and energy profiles of appliances. It provides the building blocks to describe concepts including protocols, devices, data models in the appliance domain as well as their recombination. The main element in the SAREF ontology is a Device that models objects found in homes, public buildings and offices and makes available a set of functions that are implementable through corresponding commands. Each function is in turn available on the network through a Service, whose specification includes its input and output parameters. The CoDaMOS ontology [26] models sensors that monitor a range of environmental parameters, such as moisture level, ambient light, temperature etc. Furthermore, it models actuators that are supported with semantic rule definitions specifying the control of the actuators based on the monitored environmental conditions and the presence of a person in the room. 

In [27], a survey of the existing literature is given about circular economy, summarizing the current state of the art. Be that as it may, the work on ontology for the circular economy is under-researched and there are only a few studies on this topic. The only notable work on an ontology for the circular economy is presented in [5], proposing the spatial data ontology for a textile use case that can integrate circular economy actors based on creation and post-use activities, location, and their material input and outputs. 

### 2.4. Discussion on Related Work

The current status regarding identification using contextual QR code is explained and it is transparent that another option for implementation of SmartTags is required to be able to uniquely identify tracked items and build the product passport. Our proposal is to extend the barcode by embedding additional information that could capture when a sensor changes its state. The encoded information will represent either the identity of the object or a certain sensed value, or both. Printed sensors are technology in development, and work in this topic exists, but is not relevant enough to be directly used for the development of ink with characteristics suitable for SmartTags deployment. The problem of ink formulation, as well as a utilization of ink to print the QR code and embed the information, has not been specified before. 

A review of the entity, thing and data ontologies reveals that most research defines semantic models for a combination of these concepts, with the generic “thing” concept forming a root node in most models. The common theme in these models is that they enable description of everyday connected objects, the “things” in IoT, their offered functionalities, and the observations (and/or effects) they make of their surrounding environment. The SmartTag semantic model takes this as a starting point to model the SmartTag observations, the associated units of measurement and the location aspects. Further to this, there is a need to include constructs for describing SmartTags that undergo visible changes with a corresponding change in environment.

The current state of the art does not consider the concept of visible transformations in the objects in response to changing environmental conditions. From the aspect of an ontology for circular economy, having in mind that the main focus is on recycle then reuse, the implementation is still in its infancy and there are only few studies that try to specify the data exchanges between actors. The missing ontology can set an exchange medium for the CE driving the resource information and reverse flow exchange between distant industries. 

To conclude discussion on related work, encoding and decoding methods, ink formulation for printed sensors as well as ontology for such developed tags have not been done before, and need to be specified and developed.

## 3. Methodology

The SmartTag is composed of a barcode printed partially with functional ink that can be reversible or irreversible and can react upon different environmental conditions, e.g., changes its state by disappearing when certain conditions are met, e.g., temperature goes above 10 °C. A SmartTag will therefore have to encode at least two pieces of information, the identity of the item and the sensor state; and the scanning device will have to be able to read and decode both. There are two main methods of encoding an information in the SmartTag: encoding data inside the QR code data matrix and outside the data matrix. More details about this are presented in Section 3.1.3. 

### 3.1. Design and Development of Sensors Based on Functional Ink

#### 3.1.1. Sensor with Thermochromic Ink

A thermochromic ink is a type of ink that changes color with heat. This can make certain textures appear (or disappear) as soon as the label or product goes above or below a certain temperature. These temperatures can vary from −10 to +120 °C. Temperature-sensitive inks come in two varieties: reversible and irreversible. With reversible thermochromic ink, the color will revert when the temperature returns to its original level, whereas the color remains constant after a change in temperature with irreversible thermochromic ink. Thermochromic inks are not widely available for inkjet printing, that is an essential printing method when printing individual SmartTags. That is why an effort was made to formulate an inkjet printable thermochromic ink.

**Figure 3:**
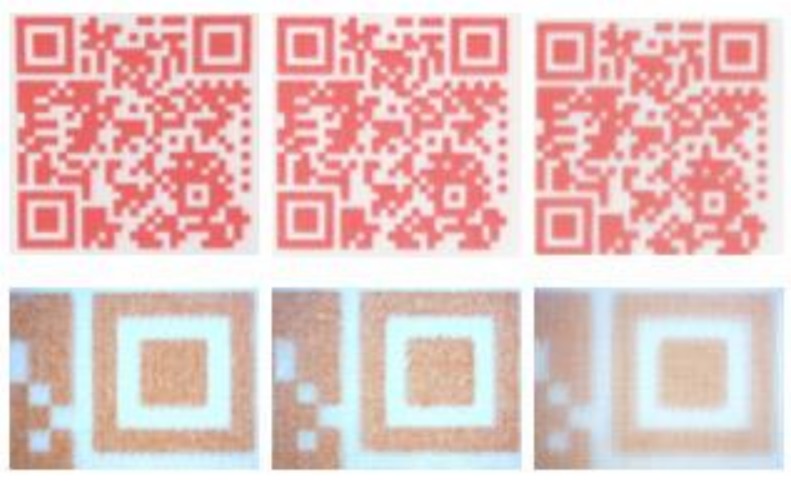
QR codes with 0.75 mm cell size printed with the thermochromic ink on different substrates: from left copy paper, label and photo paper. Pictures are taken with a camera (**above**) and with microscope (**below**).

A thermochromic ink was formulated for inkjet printing: thermochromic reversible ink changing from red to clear when temperature exceeds certain threshold (in preliminary research we did this for ink with a 47 °C threshold [28]). The colorant was thermochromic Chameleon pigment dispersion from LCR Hallcrest originally designed for flexography printing. 5 wt-% of the colorant was mixed with 1,2-propandiol, water and 0.05 wt-% of non-ionic surface active agent Dynol 604. The resulting surface tension was 33.4 mN/m and viscosity 4.75 cP, thus, making the formulation suitable for inkjet printing. For achieving dark enough codes for mobile phone reading, 10 ink layers were printed on three substrates (1) 100 g/m^2^ copy paper for color printing (copy paper), (2) label paper for laser and inkjet printing (label), and (3) 254 µm thick photographic paper from Intelicoat Technologies (photo paper). On all the substrates the QR codes with cell sizes down to 0.50 mm were successfully decoded with a camera phone (Figure 3). With this cell size, a QR code encoding the URL “http://www.tagitsmart.eu/brandprotection” has a physical size of only 14.5 mm, making it suitable for many applications where only a small area is available for the code.

#### 3.1.2. Sensors with Photochromic Ink

Photochromic materials change their color when the intensity of incoming light changes. Most photochromics change from colorless to colored upon exposure to UV light, and then fade back to colorless upon removal from the UV source [29]. The normal wavelength of excitation is around 360 nm. Commercially available photochromic dyes are usually designed as materials that can be reversibly colorized by exposure with ultraviolet (UV) light sources to a certain extent. Their usage is limited due to the lack of UV sources in the everyday environment and the destructive nature of the UV light which is revealed by the stepwise degradation of the photochromic dyes.

**Figure 4:**
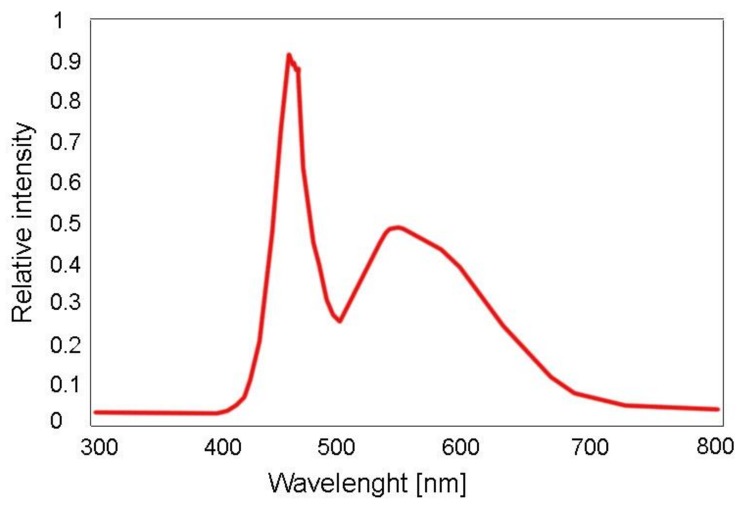
Emission spectrum of a commercial white light LED of GaN, InGaN and Ce:YAG kind for the reversible colorization of photochromic textures of this work, adapted from [30].

Herein, cyan, yellow and magenta photochromic colorant systems were developed that are reversibly colorize-able by visible light (VIS) of white light LED (W-LED) sources of GaN, InGaN, Ce:YAG kind, existent in commercially available smartphone devices. In Figure 4, a typical emission spectrum of a white light LED, preferably used as a light source for the reversible color switch of the W-LED photochromic prints of this work, is depicted.

The colorant system is soluble in polar organic fluid vehicles like cyclohexanone and tetrahydrofurfuryl acrylate. An effective colorization/decolorization is achieved at concentration of 5% to 10% by weight of the whole fluid formulation. The nature of the colorant system is monomeric; no mentionable influence of the colorant system on the overall fluid viscosity of the formulation is achieved. Therefore, its usage field is broad, and the colorant system can facilely be formulated into multiple printing fluids, for example flexographic and offset printing inks, inkjet inks, spray and roller-able inks. 

**Figure 5:**
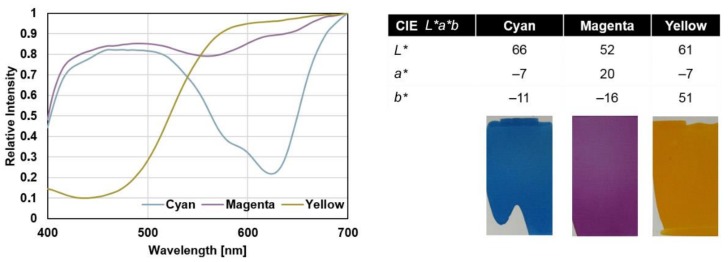
Transmission spectra, CIE L*a*b* values from the reflection UV-VIS spectra and photographs of the visible light (VIS) trigger-able photochromic colorant systems. Solvent-based ink formulations were used. The prints were prepared on plastic STYRENE sheets by doctor blading (12 µm) and drying at ambient conditions.

Characterization of the color of the VIS-trigger-able photochromic colorant systems was done by UV-VIS transmission and reflection spectroscopy. The results of the color inspections of 12 µm layers of 5 wt-% cyan (C), magenta (M) and yellow (Y) ink systems and photographs of activated prints are depicted in Figure 5.

**Figure 6:**
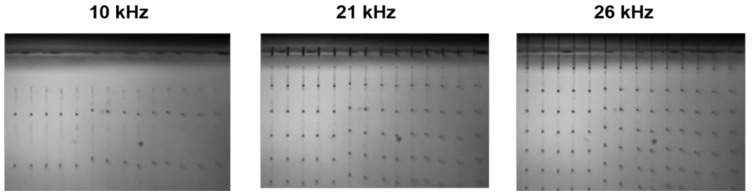
Jetting experiments of the solvent-based VIS-photochromic inkjet ink at 10, 21 and 26 kHz using an industrial inkjet printhead at 35 °C.

Formulation of the VIS-trigger-able photochromic colorant system into an inkjet-able solvent-based fluid was performed. Stable inkjet printing of the fluid was achieved at frequencies up to 26 kHz at nominal adjustable drop speeds between 6.5 and 8.5 m/s (Figure 6).

For characterization of the color, the CIE L*a*b* color space was used [31]—colored spots were measured via reflectance UV-VIS spectroscopy and the results were transferred into color values L*, the lightness, a*, the red-green value and b*, the blue-yellow value. Color differences CIEΔE(1976) as used in this work are gained by equation:

$$\mathrm{CIE}\Delta E\left(1976\right)={\left({{(L}_{colored\;spot}^{*}-L_{background}^{*})}^{2}{+(a}_{colored\;spot}^{*}-a_{background}^{*}{)}^{2}{+(b}_{colored\;spot}^{*}-b_{background}^{*}{)}^{2}\right)}^{0.5}$$

Relative CIEΔE(1976) values used as follows are defined as color differences within a series of ΔE values with regards to the maximum ΔE.

**Figure 7:**
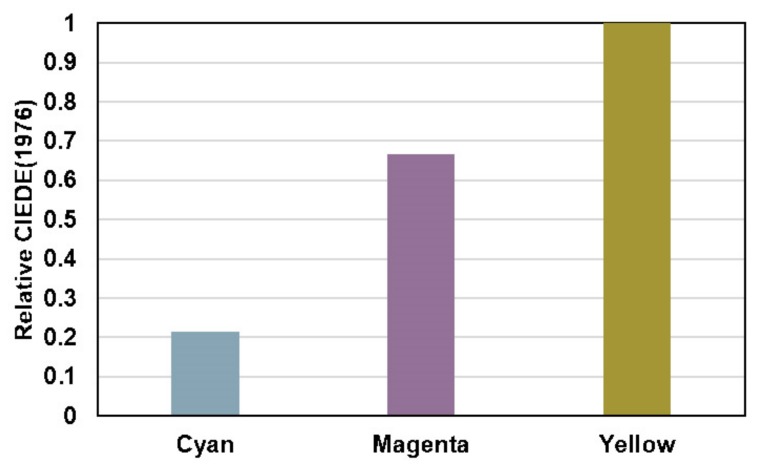
Relative CIEΔE(1976) of single colored spots of C, M and Y VIS-trigger-able photochromic 12 µm prints (5 wt-% colorant system content) in contrast to the background (STYRENE plastic sheets).

Activation experiments were performed using the W-LED of a common Samsung S4 mini Gt I9195 smartphone device. After 3 s illumination of single spots at the C, M and Y printed 12 µm layers, yellow provided the most effective colorization properties as indicated by the relative CIEΔE(1976) values of the activated spots in contrast to the non-colored background (Figure 7).

**Figure 8:**
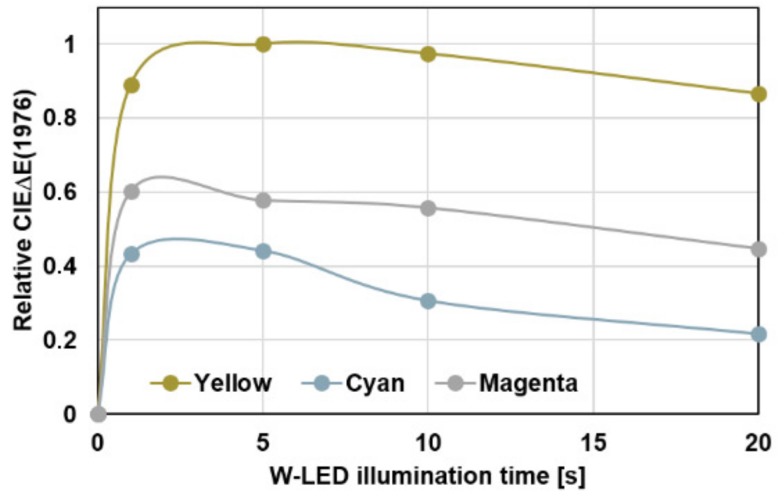
Colorization of C, M and Y VIS-trigger-able photochromic printing layers as a function of the illumination duration using the white light LED (W-LED) light source of the Samsung S4 mini smartphone device.

An optimum colorization of the VIS-trigger-able photochromic prints can be achieved by illuminating the single spots for 3–5 s (Figure 8).

**Figure 9:**
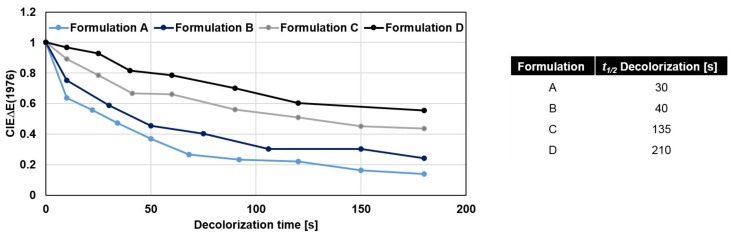
Decolorization dynamics and half-life periods *t*1/2 of decolorization of yellow VIS-trigger-able photochromic 12 µm prints of formulations A, B, C and D by varying the reactant ratio of the colorant system. Relative CIEΔE(1976) values are relative color differences with regards to the maximum color difference within a single series which is the color difference directly after the illumination period at *t* = 0 s.

The decolorization dynamics of yellow VIS-trigger-able photochromic prints by varying the reactant ratio of the colorant system are depicted in Figure 9.

The decolorization dynamics of the photochromic prints are adjustable by this method as indicated by the half-life period *t*1/2 of decolorization with regards to the background color. 

#### 3.1.3. SmartTags Data Encoding

**Figure 10:**
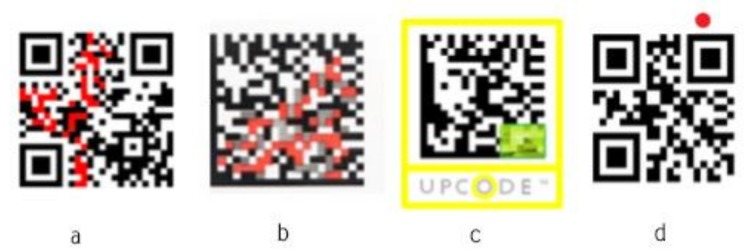
Types of sensors encoding in the SmartTag: (**a**) encoding inside the QR code matrix with one ink; (**b**) encoding multiple types of functional ink (grey and red); (**c**) encoding sensor (green) as a part of QR code not related to the data matrix code; (**d**) encoding sensor outside of the QR code matrix (red).

The SmartTags have to encode at least two pieces of information, the identity of the item and the sensor state. Depending on how these data are embedded in the SmartTag, different encoding should be applied, defining how the information about the identity and the sensing capabilities are going to be represented in the SmartTag. There are two approaches to build the SmartTag from QR code and functional ink:

1. To encode functional ink as a part of the QR code data matrix to initiate change of the content when ink appears or disappears (Figure 10a,b); in further text referred to as “Type 1.”
2. To print functional ink over or outside the QR code data matrix not influencing any data of the data matrix (Figure 10c,d); in further text referred to as “Type 2”.

The benefit of the first over second approach is the possibility to use a standard QR code scanner. The second approach will require manual development of the reader that will be capable to decode change of the sensor state, i.e., to detect appearing context, i.e., letter, square, line or something else. 

**Figure 11:**
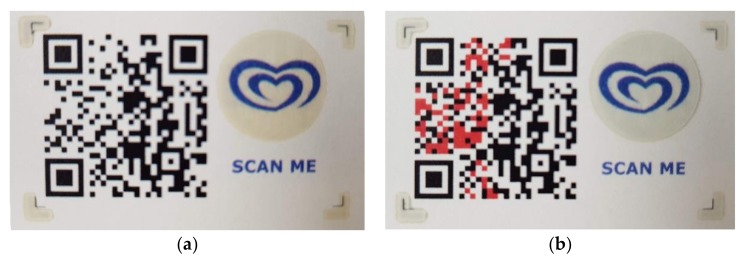
QR code before (**a**) and after (**b**) sensor state change encoded with two URLs with changed last two characters.

We will focus in further text of this subsection on the first SmartTag encoding approach, i.e., encoding sensors as a part of the QR code matrix using one ink. The second approach is straightforward, and encoding is not necessary. The methodology will be explained in an example of encoding URL with parameters inside the QR code. Having in mind that this feature was not foreseen with the original QR code (2D barcode) standard, a searching algorithm was developed that generates two QR codes which differences are not overlapping. The algorithm is developed with following logic:

1. Generate reference QR code (in this case Figure 11b.)
  a. Content type: URL
  b. Data: e.g., “https://zfrdd.tn.gg/gtin/8712100836275?UD”
  c. Error correction: Low
2. Generate batch of QR codes with changed last two characters (URL parameter part—“UD” in this case); again new characters, if letters, must be capital
3. Compare each generated QR code from batch with the QR code generated at step (1) and find the QR code which differences are not overlapping
4. Algorithm found non-overlapping QR code
  a. Data: “https://zfrdd.tn.gg/gtin/8712100836275?S0”
5. Make “mask” from difference between (1) and (4) that will be used for printing over reference QR code (1)

In this example, the algorithm found that the QR code with parameter “S0” does not overlap and the data matrix difference of this QR code will be extracted and printed with functional ink over reference QR code. The main requirement is that changing letters in URL are capital, and that the length of both strings is the same to ensure that the amount of changing fields is as small as possible. 

The limitation is that there is a final number of QR codes that can be generated using this method. Changing the error correction value and the percentage of points that can be removed, the number of QR codes that can be generated could be increased to some extent. This approach is viable for both SmartTags with appearing and disappearing ink. The version with two inks in the data matrix uses the same methodology described above, but it requires generation of additional QR codes and repeating of step (3) until three QR codes are found with non-overlapping differences.

The tags encoded in SmartTag do not have an interface for connecting to the Internet and the connection is actually established during the scanning process in which a mobile or scanner device acts as a gateway by triggering a different URL (sensors activation will change the embedded information in the QR code). 

### 3.2. Sensor Evaluation Methodology

The goal of the evaluation of the printed sensors was to justify if the proposed colorant system for photochromic and thermochromic ink is working under predefined parameters, i.e., if the exposure to a predefined amount of light and temperature will trigger expected changes in ink. Accordingly, sensors engineered, taking into account different colorant systems, were developed and evaluated first in laboratory environments, until a certain performance is satisfied. After having a first version of printed sensors stable and reacting as predefined during exposure on different environmental parameters, the evaluation of printed sensors was done by evaluating Type 1 and Type 2 tags’ ink ability to be recognized/decoded with a standard QR code scanner and image vision respectively. The performance evaluation was facilitated over four different use cases leveraging available inks, evaluating in total two types of inks: thermochromic (reversible and irreversible) and photochromic (reversible).

## 4. Performance Evaluation

### 4.1. Thermochromic Ink

In the preliminary evaluation [28], sensors were evaluated and calibrated by having a SmartTag with a sensor cooled down and warmed up in a controlled environment. During these tests, environmental variations were introduced, i.e., illumination variations, shadows and varying the light source color and intensity. From this preliminary testing, sensor values were analyzed against the threshold and evaluated if the ink changed according to predefined values of temperature in environment. Taking into account data collected (i.e., sensors reactions, annotations on sensor visibility and reactions) calibration of the ink was done. The ink calibration itself is out of scope of this research and will not be explained.

#### 4.1.1. Sensors in Data Matrix: Thermochromic Ink Evaluation 

**Figure 12:**
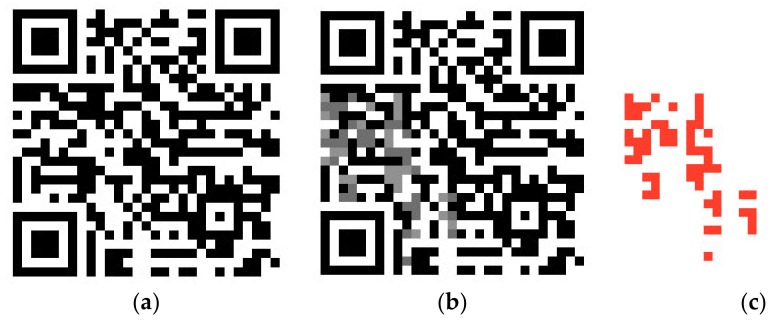
Example of SmartTag with irreversible thermochromic sensors used in the evaluation: (**a**) Reference QR code before changing state; (**b**) QR after sensor change state and marked difference between data matrixes (grey); (**c**) functional ink for printing used as a mask over reference QR code.

After successful laboratory testing and proof of concept, sensors were encoded in the QR code matrix (SmartTag Type 1) and evaluated for readability when exposed to predefined environmental conditions. In order to evaluate if QR code could be successfully decoded, the evaluation was executed using irreversible thermochromic tags in ice cream use case. In total 150 SmartTags was evaluated by exposing the tag to temperatures greater than –10 °C and scanning the tag when the ink changes. From 50 tags, three tags were not readable as the color was too pale due to the bad printing, probably during lowering the amount of ink in the cartridge. The example of QR code used in the evaluation is presented in Figure 12, together with the QR code after sensor state change (Figure 12b) and mask used for printing (Figure 12c). 

#### 4.1.2. Sensors over Data Matrix: Thermochromic Ink Evaluation

**Figure 13:**
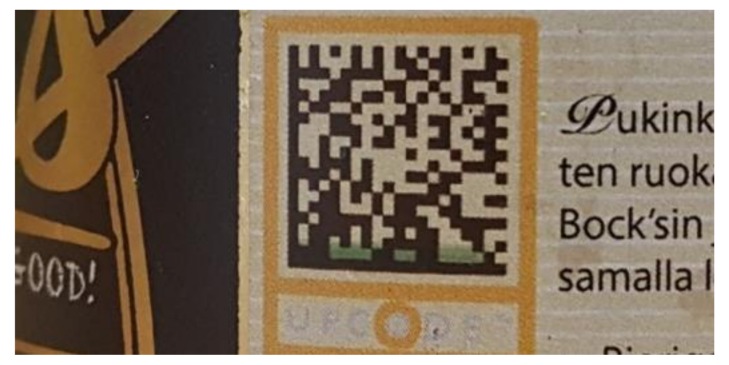
SmartTag with reversible thermochromic sensors used in the evaluation.

The evaluation of the reversible thermochromic ink applied over QR code (Type 2c) was done in the beer use case, labeling bottles with SmartTags that activates and becomes visible when the temperature is lower than 8 °C, as shown in Figure 13. A total of 50 SmartTags was evaluated by exposing the tag to temperatures greater than 8 °C and by scanning the tag when the ink changes. From 50 tags, all were readable before and after the sensor changed its state. Note that the vision algorithm is implemented to detect green color, and that the data matrix does not change. The algorithm was decoding the QR code and detecting green color at the same time.

#### 4.1.3. Sensor Outside QR Code: Thermochromic Ink

**Figure 14:**
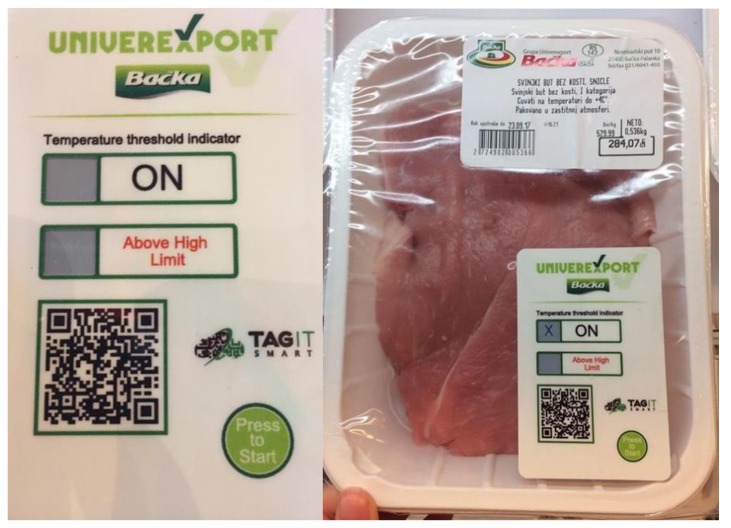
SmartTag with irreversible thermochromic sensor used in evaluation.

The irreversible thermochromic ink applied outside QR code (Type 2b) was evaluated with a meat use case by monitoring the temperature over 7 °C. The total 50 SmartTags were printed with irreversible ink over the grey box to the left of “Above High Limit” (Figure 14) in which ink appears as an “x” character after the sensor reacts. The detection of appearing ink is done using the image vision algorithm and from a total of 50 tags, all were readable. 

### 4.2. Photochromic Ink

**Figure 15:**
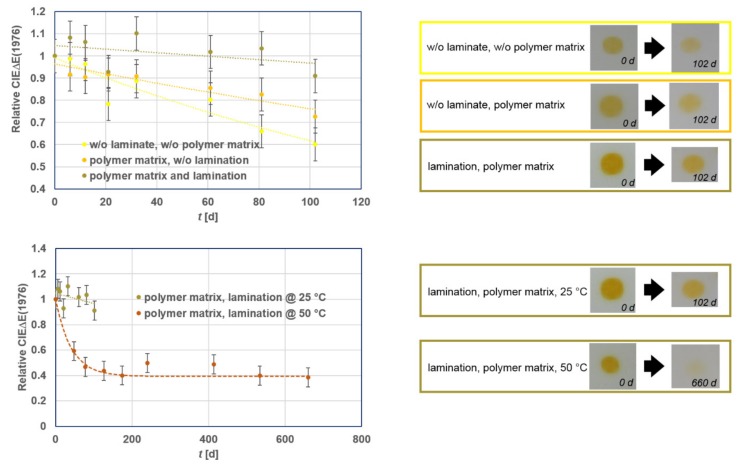
Top: Colorization performance of yellow VIS-trigger-able photochromic 12 µm print layers with varying stabilization technology at 25 °C within a period of 102 days of observation. Bottom: Colorization performance of the most durable yellow VIS-trigger-able photochromic stabilized colorant system at 25 and 50 °C.

Sensitivity of the colorant system versus elevated temperatures and oxygen were first evaluated in laboratory settings. This was done by monitoring the durability of the prints at 25 and 50 °C. Prints were created on a label-material and different protection strategies were applied (Figure 15).

When the sensors are printed solely using the colorant system, after 102 days of evaluation at 25 °C, the colorization durability was significantly dropping (the color sharpness was 60% lower), i.e., the print appears pale yellow. A significant enhancement of the colorization durability was achieved by incorporating the colorant system into a polymer matrix that is present within the ink formulation (ΔEdrop, 102 d = 30%). The most efficient stabilization of the VIS-photochromic system was achieved by both incorporating the colorant system into a polymer matrix that is provided within the ink formulation and laminating the prints with a transparent self-adhesive foil. In this setup, solely a drop in the relative CIEΔE(1976) by 10% was detected after 102 days at 25 °C. 

A massive drop in durability of the laminated, and in a polymer matrix incorporated yellow VIS-photochromic colorant system, was detected when storing the prints at 50 °C (Figure 15, bottom). Activation of the 50 °C sample by a 3 s W-LED illumination was barely possible after 102 days whereas the sample stored at ambient conditions was almost as intact as at the beginning of the experiment.

Considering the above mentioned stabilization technology, a proper shielding of the colorant system by its incorporation within a polymer matrix, lamination of the prints, and handling and storing of the prints at ≤25 °C, are the key factors for their optimum durability. 

**Figure 16:**
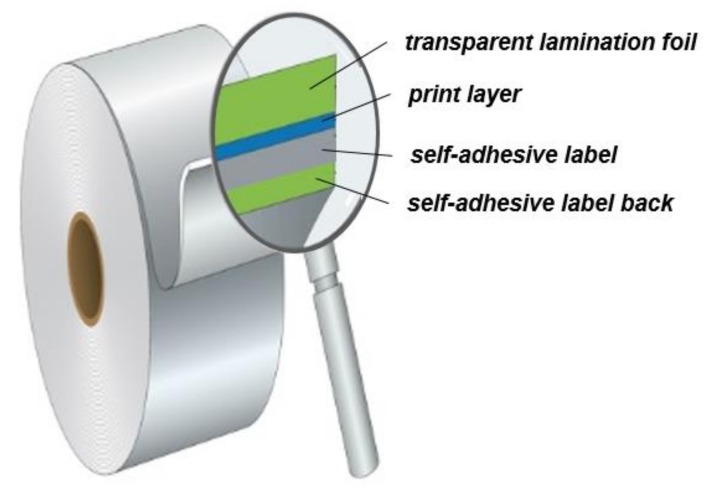
Conceptual schematic on the architecture of a R2R (Rool to Rool) media containing VIS-trigger-able photochromic prints of this work. The functional inks can be printed by any method on self-adhesive R2R-media. The photochromic colorant system is embedded within a polymer matrix that is existent in the ink formulation. In a subsequent step, the prints are covered by a self-adhesive transparent lamination foil to achieve optimum durability of the photochromic prints.

A realization of this concept on an industrial scale was performed on a flexible self-adhesive R2R (Rool to Rool) label material as schematically depicted in Figure 16. After printing the VIS-trigger-able photochromic ink on a polypropylene-based label material, lamination foil was applied on top of the prints.

#### Sensor Outside Data Matrix: Photochromic Ink Evaluation 

The evaluation of the reversible photochromic ink applied outside the QR code (Type 2) is done by scanning of photochromic tags that react to a mobile device’s LED flashlight. This part of the SmartTag contains photochromic letter, visible only after exposure to light. The algorithm is developed that detects letter that appears after exposing the SmartTag with light. The number of used tags in evaluation was 100, and the readability was 85%. 

**Figure 17:**
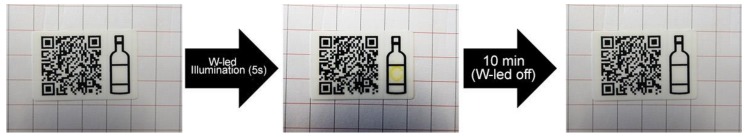
Activation of a SmartTag based on reversible W-LED-sensitive photochromic sensor.

In Figure 17, an example of a SmartTag printed with photochromic ink is given. When the SmartTag is introduced to direct contact to light and illuminated for 5 s, the texture “C” appears; e.g., in this case a standard handheld device white light LED from camera flashlight was used. The “C” disappears within 10 min in absence of the flashlight. The process is reversible. When the colorization gets back to its original stage, multiple readouts of the tag is possible.

### 4.3. Results

The evaluation results of decoding capability of SmartTag printed with functional ink for two categorized encoding types showed that encoding data inside the QR code data matrix (Type 1) has certain restrictions and limitations: (1) the number of QR codes that can be generated using this method is final and (2) the readability of the QR code drops with the density of ink, influencing QR code decoding ability.

The decoding of ink printed over the data matrix and outside the data matrix (Type 2) showed good performance and the readability was excellent for the thermochromic ink, while photochromic ink was not readable in 15% of cases out of 100. Accordingly, from laboratory testing and evaluation it can be concluded that photochromic ink has certain limitations of: (1) it can be used in a scenario where environmental temperature will not go above 25 °C; (2) approximately 15% of failed scans could appear due to the loss of coloration intensity of ink. Colorant intensity, to some extent, can be compensated when the scanning process is done with a more powerful device, but in a scenario when the user randomly introduces their device, the scanning could fail. If a proper shielding of the colorant system is done, as explained in Section 4.2, and if prints are used in temperatures lower than 25 °C, the optimum durability can be achieved. The irreversible photochromic ink was not evaluated in this research; thus, this can be interesting for future work. From our previous experience, it can be expected that similar results would be achieved with irreversible as with the reversible photochromic sensors.

Photochromic yellow ink cannot be used for Type 1, i.e., for printing the QR code data matrix, as it would be to pale for the scanner to detect it. Instead, we have printed it outside of the QR code and detected if ink appears using image vision algorithms. It should be noted that the goal of the evaluation was not to evaluate if ink is reacting on predefined environmental conditions, but to evaluate the ability to decode data from the SmartTag after the sensor reacts. All results from the performance evaluation are presented in the Table 1, including SmartTag type and ink type, activation parameter, number of tags, as well as percentage of successful decoding. 

## 5. SmartTags for Circular Economy

In this section, different methods for SmartTag encoding of sensors in the QR code are explained to enable embedding of data inside the QR code data matrix after sensors have changed its state, i.e., ink has appeared or disappeared depending on the type of ink used to build the SmartTag. After encoding, the tag can be used (tagged) in different scenarios and the extracted data from sensors used to achieve better understanding of how, where, and when some entity is being used; providing additional data for the product passport. 

Circular economy is driven by the possibility to exchange the data about the product passport, which is not possible if there is no specified ontology. Therefore, the SmartTag’s ontology as well as linked spatial data as a common ontology for connecting product passports and integrating ecosystem actors based on their input and output material as a main requirement is provided. The CE ontology proposal was relying on [5]—we have specified SmartTag ontology first from scratch based on more than 10 end user pilots from the TagItSmart project [32]; afterwards, we have mapped it with [5] to provide more unified work on Circular Economy ontology. 

### 5.1. Ontology and Semantic Model

Since the circular economy is predicated on cycling products and materials across different value streams, descriptions of the materials’ composition, condition, reuse and recycling potential are key [5]. Information about the provenance about the product also needs to be factored into the product passport, along with a strong spatial awareness to allow the various players to engage in circular exchanges. 

#### 5.1.1. Circular Economy Ontology

This section first presents a high-level view of the core set of data models defined to describe the various players in the CE, which include the smart object, i.e., the physical entity and its SmartTag enabled digital version, i.e., virtual entity (VE), users (which can include companies manufacturing the SmartTag-enabled products or recycling/dis-assembling/reusing them), services as well as the SmartTag. 

The models are implemented using semantic Web technologies (i.e., Web Ontology Language (OWL)) in order to cope with the challenging problems of heterogeneity and interoperability caused by the large number of objects with different characteristics. Following the Web of Data’s Linked Data principles, the models are not defined in isolation, but include properties that allow linking to each other (where appropriate) and also to external domain ontologies; for instance, the global location URI of an entity could link to the relevant location instance in the GeoNames ontology, where the given location is more fully described.

**Figure 18:**
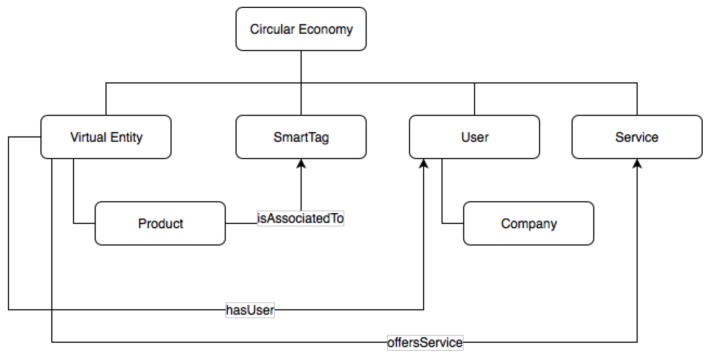
Circular Economy concepts and data.

Figure 18 below presents the exchange relationship between distinct economic actors in the CE. Products, which are specializations of the VE concept, are associated to SmartTags, which provide identity and environmental condition information. Each VE concept is linked to different Users during its lifecycle, which could represent a person as an owner of the VE or a company that created the product. User characteristics described in the user ontology include first, given or family names, age, gender, and preferred language (as a 2-letter code). In addition to the geographical coordinates as defined in the VE model, the user model’s Location definition also specifies the county and country IDs in terms of the NUTS classification [33]. A user can have different roles that are associated with particular access rights and can describe varying ownership of the product during its lifecycle. 

Services are linked to VEs and encapsulate creation and post-use activities, which each having specific inputs and outputs. The following sections detail the individual data models.

#### 5.1.2. Virtual Entity and Service Ontology

**Figure 19:**
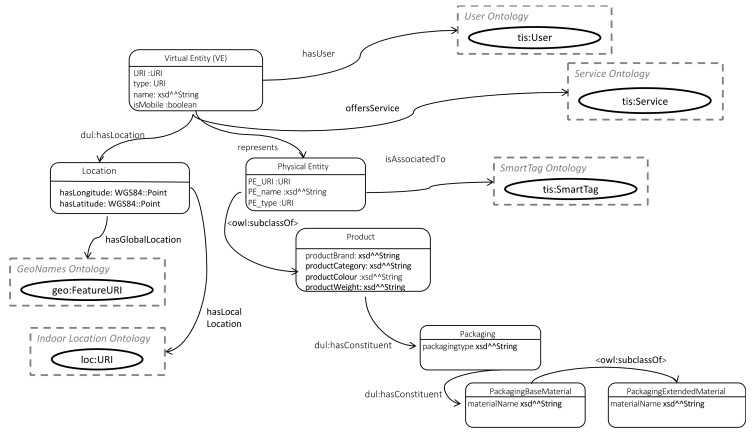
Virtual Entity ontology.

This section first details the VE ontology classes and properties, as shown in Figure 19. Following the ontology design principles of reusing and extending existing ontologies, the VE class is defined as a sub-class of the dul:Entity class. As such, it inherits a number of properties from the dul:Entity class, such as *hasLocation*, *hasConstituent*, *hasPart* and *isDescribedBy*. 

Since the VE is a digital abstract representation of a physical entity, this is represented through the *represents* property linking the VE and Physical Entity (PE) classes. Similar to the VE class, the PE is a sub-class of the dul:Entity class and is defined as either a dul:PhysicalAgent (e.g., a Person or an Organism) or a dul:PhysicalArtifact (e.g., natural objects in the CE with a certain function, that can be recycled, or structurally designed objects), expressed through the ∪ (unionOf) operator. This is shown in DL notation in (1) below:

(1) Physical_Entity≡PhysicalAgent∪PhysicalArtifact.

To meet the specific requirements of the CE, a specialised type of PE is defined, i.e., a Product, which is defined as a sub-class of the PE class. Since the circular economy requires knowledge of the product materials, a Packaging class is defined. The inherited dul:*hasConstituent* property for the *Product* class, is refined by placing an existential quantifier restriction (Ǝ) on its range to the *Packaging* class, as shown in (2):

(2) Product.dul:hasConstituent ∃ Packaging.

The Packaging class is defined by its type (e.g., bottle, film, label etc.) and by constraining the inherited *dul*:*hasConstituent* property to instances of the PackagingBaseMaterial. The *PackagingBaseMaterial* class has a sub-class, the *PackagingExtendedMaterial* class, to enable more specialised annotations of the packaging materials. An example could include plastic as the base material and “grade 5 plastic PP” as the extended packaging material. Together, the Product and Packaging classes capture information related to the product’s qualities, which can guide its recycling and other post-use activities.

The VE location, is specified through the Location class, to which a VE is linked to using the inherited *dul:hasLocation* property. The Location class is specified through a number of properties which represent locations at different granularities. These properties include latitude (*hasLatitude*) and longitude (*hasLongitude*), which are represented in terms of the WGS84 Point specification [34]. Where available, the VE could also have a global location (*hasGlobalLocation*) specified by linking to an instance in the GeoNames ontology. At a more granular level, the location could also be specified in terms of relative location within an indoor location ontology through the *hasLocalLocation* property.

**Figure 20:**
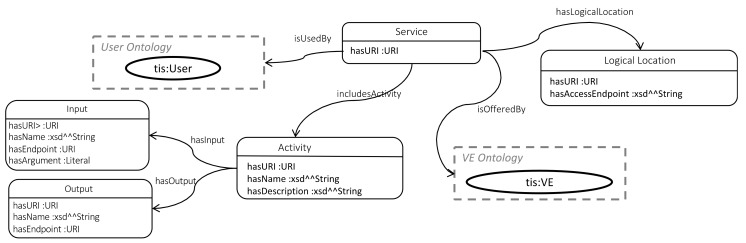
Service ontology.

The Service ontology, shown in Figure 20, is designed to describe the creation and post-use activities of the SmartTags as well as the VEs in the CE. Each service instance has a unique identifier (*hasURI*) specified by a URI and can be part of a VE’s functionalities, as specified by linking it through the *isOfferedBy* object property to an instance of the VE model. The user of a service is described through the *isUsedBy* object property associating a service instance to an instance of the User model. This can be used to describe the creation or post-use activities (that are part of the service) undertaken by the User instance. 

The possible activities are specified in terms of accepted inputs and outputs. Both the Input and Output class include an URI, type (e.g., to specify the semantic type), name and endpoint specification (e.g., to specify the endpoint URLs through they are accessible). In addition, the Input class also has an argument property (*hasArgument*) to specify the input arguments that the function accepts. 

The service class also has a property (*hasLogicalLocation*) linking it to the logical location of the service (*LogicalLocation* class), which could be defined as the URL of the software implementation of the service, such as some endpoint, complemented by a particular set of properties provided by some API for the service.

#### 5.1.3. SmartTag Ontology

**Figure 21:**
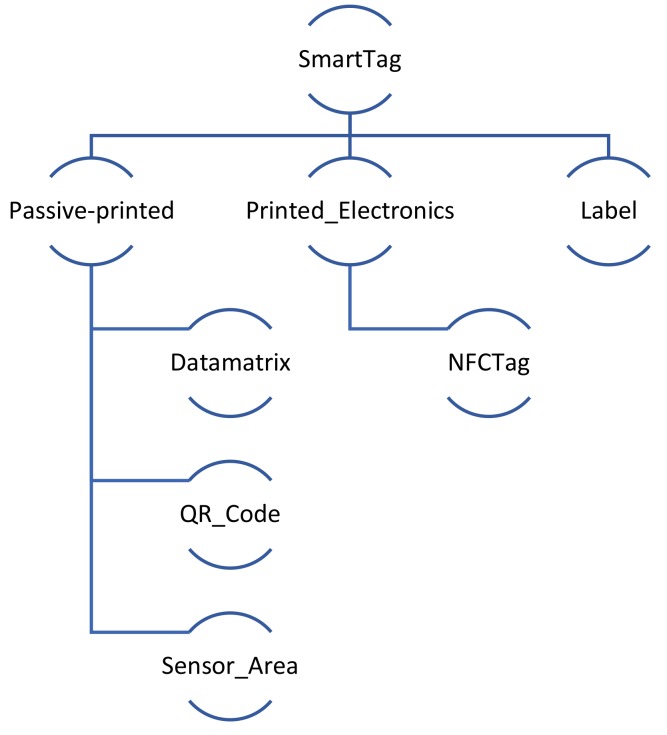
Top-level hierarchy for the SmartTag.

**Figure 22:**
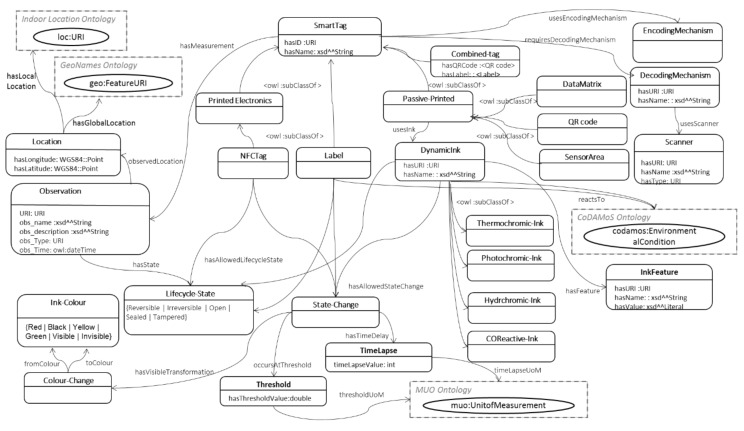
Semantic Ontology for the SmartTag.

This section details the SmartTag model that captures the environment-reactive properties of both passive printed 2-dimensional tags (e.g., QR codes and datamatrix), electronic tags (e.g., NFC) and Labels, e.g., time-indicator or temperature-time-indicator label, and a Sensor_Area specification, which depicts an area printed below a standard QR code, with functional inks that react to changing environmental conditions. The resulting top-level hierarchy of the SmartTags is as shown in Figure 21 below and the resultant SmartTag model is depicted in Figure 22. 

The information embedded in the SmartTags can be retrieved by applying the appropriate decoding mechanism, as specified through the *requiresDecodingMechanism* object property to the *DecodingMechanism* class. Each passive printed SmartTag also features an encoding mechanism that is described in the *EncodingMechanism* class.

Each SmartTag maintains a record of the scanned measurements by instantiating an object of the *Observation* class, which includes a literal name (*obs_name*), *URI* (linking to an external domain model instance that specifies the environmental feature being observed, e.g., temperature), (string) *description* (what the observation is about), type (*obs_Type*) and the time of the scan (*obs_Time* property specified in terms of an owl:dateTime instance). Each observation record also includes location context information. Each observation also captures the lifecycle state of the SmartTag, through the LifecycleState class. The LifecycleState class is defined as an enumerated class, which implies that it is a class of the instances, (and only the instances) listed in the enumeration. In this case, the allowed states and hence the instances of the class are: Reversible | Irreversible | Open | Sealed | Tampered. The first two instances refer to the allowed states for dynamic inks and the latter three to those for NFC tags.

(3) LifecycleState≡{Reversible Irreversible Open Sealed Tampered}.

As the passive tags can be encoded using functional inks that react dynamically to changing environmental conditions, the related properties are captured in the *DynamicInk* class. Each *DynamicInk* class instance is described using the ink features, which could include reaction times, shelf life etc. specification for the corresponding ink. The environment feature that these different inks respond to is described by the *reactsTo* property and by placing relevant restrictions on the property range depending on the sub-class. The thermochromic ink sub-class’s *reactsTo* property specification is defined by:

(4) Thermochromic−Ink.reactsTo ∀ codamos:Temperature.

An important aspect of the inks is the color and visibility changes at certain environmental condition changes. This is described through the *State-Change* class, which is linked to the *DynamicInk* class through the *hasAllowedStateChange* property. The visible transformations inherent in a state change are defined by linking the *hasVisibleTransformation* property from the *State-Change* class to the *Color-Change* class. The *Color-Change* class describes 2 properties: *fromColor* and *toColor*, with both property ranges defined as instances of the *Ink-Color* class:

(5) Ink−Color≡{Red Green Yellow Black Visible Invisible}.

The threshold value at which the color (or visibility) change occurs is described through the *occursAtThreshold* property to the *Threshold* class, which in turn defines a threshold value (*hasThresholdValue*) (as a double) and an associated unit of measurement (*thresholdUoM*) defined in terms of an instance of the MUO ontology:

(6) State−Change.occursAtThreshold ∀ Threshold.

The time delay information is described with the *hasTimeDelay* property between the *State-Change* and the *TimeLapse* class. The *TimeLapse* class has a timelapse value (*timelapseValue* as an integer) and an associated unit of measurement (*timeLapseUoM*). The values for the unit of measurement are constrained to instances of the MUO ontology [35] by the existential quantifier:

(7) Time−Lapse.timeLapseUoM ∃ muo:UnitofMeasurement.

The Label class has been defined as a sub-class of the root SmartTag class. In addition to the tag ID, name and size specification (the TagSize class and the corresponding properties have not been shown in Figure 22 due to space restrictions), it also specifies the lifecycle state attached with the label (reversible/irreversible). In addition to the already defined properties, it captures the threshold value (if any) at which the state transformation is triggered, the visible (color) transformation involved and any optional time lapse indications if associated to the state change. Together, these three conditions allow capturing a range of different combinations of the possible labels that can be encountered in the real-world examples, including time indicator labels (which only have the time-lapse value of the state change) and temperature-time indicator labels (which have both time-lapse and threshold combined with color change specifications). 

For any SmartTag having some measure of time lapse involved, an additional *activationTime* property has been introduced, which captures the date and time at which the time indicator is activated. This property is associated with the *TimeLapse* class, with *xsd:dateTime* as its range.

The Sensor_Area class has been defined to depict a patch printed using functional inks (thermochromic or hydrochromic etc.) to show visible transformations as a response to the corresponding environmental condition, e.g., a sensor area printed using hydrochromic ink reacts to the presence of humidity/water. Therefore, the Sensor_Area class has been defined as a sub-class of the *Passive-Printed* class and inherits all the properties and restrictions of its parent class.

### 5.2. Demonstration of the Printed Sensors Ontology and Semantic Model

**Figure 23:**
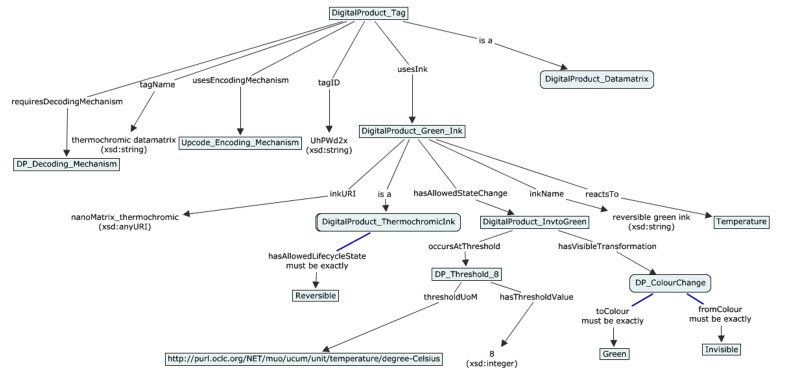
Semantic model for digital product use case with beer.

**Figure 24:**
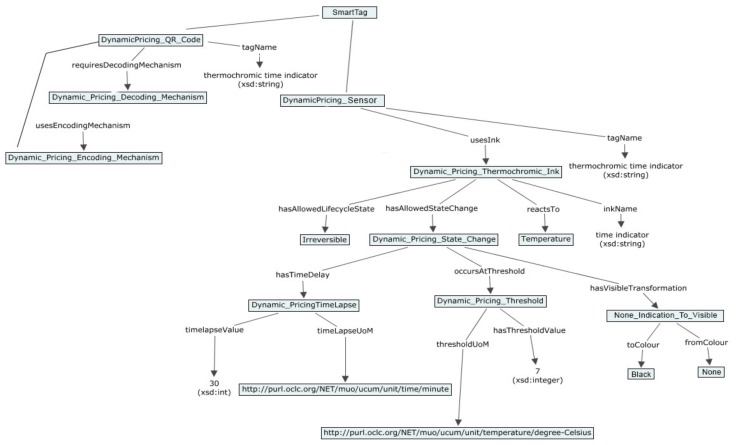
Semantic model for use case with meat.

**Figure 25:**
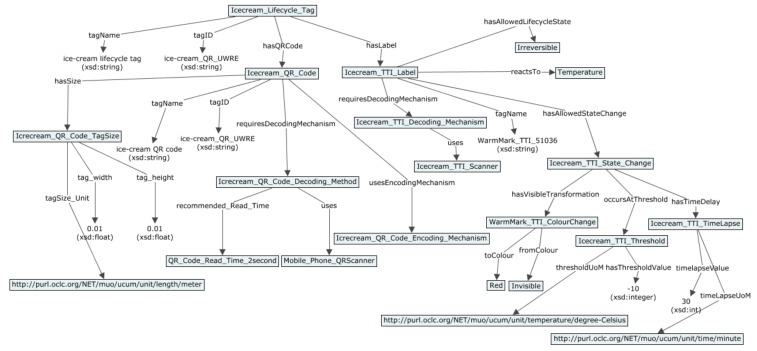
Semantic model for ice cream use case.

**Figure 26:**
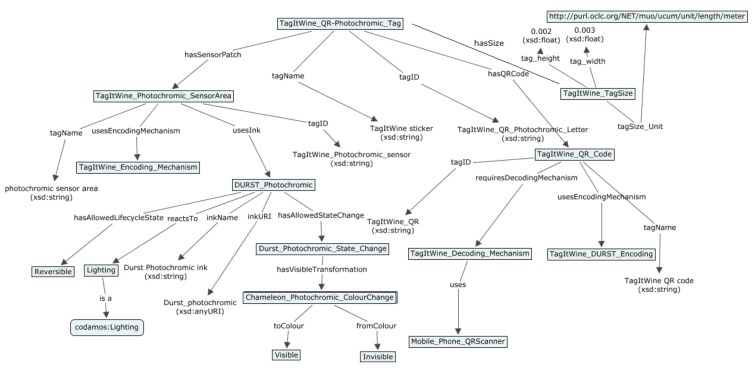
Semantic model for wine use case.

In this section, the example of the semantic model for SmartTag with printed sensors using use case scenarios from Section 4 is provided. In Figure 23, Figure 24, Figure 25 and Figure 26 semantic model for digital product use case with beer, meat, ice cream and vine are shown respectively as an example of how the SmartTag ontology could be drafted using the proposed approach. 

## 6. Discussion

The proposed SmartTag, its semantic model, and ontology goes beyond the state of the art as it includes constructs for developing QR codes that undergo visible changes with a corresponding change in an environment feature (e.g., temperature, light) near a threshold level. The model also enables error checks for observations made for these visible changes by facilitating its association with the stated ink technology’s reversible/irreversible state.

The benefit of product lifecycle and data exchange is the ability to collect a set of information beyond available data (i.e., components and materials that product contains), enhancing the level of details about the product passport including when it is being used, how, and by who; environmental conditions, etc. Semantic ontology is practically enabled for these data exchanges that can be used as an input to build such exchange mediums. SmartTags composed of functional ink can measure parameters of the physical environment, capturing more data than possible in the moment with current technology without using expensive tracking and monitoring mechanisms. 

Data provided using tags will give an overview of the used materials, their recyclability and potential for reuse, and a value model can be created that allows all stakeholders—from factory to recycling, from producer to individual customer—to not only track and trace any item in the process, but also to provide additional information about the item that can be of use. This includes giving individual feedback to a producer, requesting a new product based on individual taste and alerting retailers and producers that an item is close to its best before date or that it is being disposed of (in a recycling bin) as it should. The reuse of material and improved resource efficiency might have a positive impact on circular economy, lowering waste disposal and offering companies to take obsolete products and make new ones.

Further to this, additional information for product passport will set collection, refurbish, repair, reuse and recycling infrastructure in place offering mechanisms:

- To enforce product standards and legislations for waste management and reuse and recycling
- For provisioning and exchanging of information on availability of reusable or recyclable materials when a product becomes obsolete, information to manufacturer about durability, quality, etc.

## 7. Conclusions

In this study, we have presented SmartTags technology for provisioning of the unique item-level identities. Printed temperature and luminosity sensors were used for creation of the dynamic QR codes. The ink formulation is explained for photochromic and thermochromic types of ink, targeting different use cases mostly related to products for mass consumption, but its use is not strictly restricted to these. This research has showed that SmartTags can be used in circular economy for unique item-level identification and detection of environmental parameters in which a product is kept, transported or exposed with certain limitations. The evaluation included assessment of the scanning ability of the surface printed with functional ink after changing of sensor’s state for two cases: when ink is encoded as a part of the QR code and when the ink is printed over or outside the QR code. The limitation for encoding ink as a part of standard data matrix is the restricted number of QR codes that can be generated using this method and the drop off in readability of the QR code with the density of ink. Accordingly, it is required to provide new standards for QR codes that would take SmartTag requirements encoding as an input. This will be done in future work. The limitation for ink printed, not related to QR code matrix, is the drop in colorant intensity, which for thermochromic ink is negligible, but for photochromic ink the estimation is that 15% failed scans could appear. We should be aware that product passport goes beyond the data about product specifications: environmental conditions can be augmented with printed sensors, i.e., triggering modification of the barcode tag will allow additional data aggregation for the product passport. This is pioneering work on the topic and proposed unique item-level identifiers could be printed with ordinary ink if requirements for the functional ink are not met, i.e., if ink cannot perform well for certain parameter values. Further to this, developed technology will enable data exchange leveraging provided ontology for the circular economy.

## 8. Standardization

In this study conducted as a part of research during the H2020 TagItSmart project Grant Agreement Number 688061, during development of SmartTag technology we have followed GS1 standards for coding of packaging to implement tags with printed sensors. We have ratified in August of 2018, the new global GS1 Digital Link Standard [36] substituting the current 1D barcodes for Web based QR codes. Similar to the way a web address (URL) points to a specific website, GS1 Digital Link enables connections to all types of business-to-business and business-to-consumer information. And instead of being limited to one type of data carrier like a traditional barcode, brands can now use a QR code, radio-frequency identification (RFID), GS1 DataMatrix tag or near-field communication (NFC) to deliver this information to their customers. To help the community understand the powers of the Digital Links, we also contributed with a set of open source tools [37] that allow people to experiment with the Digital Link, independent of the platform. 

## Figures and Tables

**Table 1:** SmartTag evaluation results.

SmartTag	Use Case	Ink Type	ActivationParameter	Ink Characteristic	Number of Tags	Number of Successful Decoding
Type 1*	Ice cream	Thermochromic	Temp > −10 °C	Reversible	50	96%
Type 2	Wine	Photochromic	Lux > ~100 Lumen	Reversible	100	85%
Type 2	Beer	Thermochromic	Temp > 8 °C	Reversible	50	100%
Type 2	Meat	Thermochromic	Temp > 7 °C	Irreversible	50	100%

*Type 1—data is encoded in a standard QR code data matrix
